# The incidence and risk factors of depression across six time points in the perinatal period: a prospective study in China

**DOI:** 10.3389/fmed.2024.1407034

**Published:** 2024-08-23

**Authors:** Jue Zhu, Youchun Ye, Xuan Liu, Yichen Chen, Lu Chen, Yi Lin, Qiming Wang, Jing Zhang

**Affiliations:** ^1^Department of Gynecology and Obstetrics, Women and Children’s Hospital of Ningbo University, Ningbo, Zhejiang, China; ^2^Department of Gynecology and Obstetrics, The Affiliated People’s Hospital of Ningbo University, Ningbo, Zhejiang, China; ^3^Department of Computer and Data Engineering, NingboTech University, Ningbo, Zhejiang, China; ^4^Department of Basic Research Laboratory, Women and Children’s Hospital of Ningbo University, Ningbo, Zhejiang, China

**Keywords:** perinatal depression, incidence, risk factors, six time points, Edinburgh Postnatal Depression Scale

## Abstract

**Introduction:**

Perinatal depression (PND) affects approximately 15%–20% of women. This study aimed to determine the incidence of PND and identify risk factors.

**Methods:**

A prospective study was conducted at the Affiliated People’s Hospital of Ningbo University. The Edinburgh Postnatal Depression Scale (EPDS) was used to screen for PND. Classification models were constructed using Extreme Gradient Boosting (XGBoost), Logistic Regression (LR), Random Forest (RF), and Support Vector Machine (SVM), and the optimal model was selected.

**Results:**

Between March 2019 and August 2021, a total of 485 participants completed all valid questionnaires. Depression was observed in 75 (15.5%), 47 (9.7%), 25 (5.2%), 94 (19.4%), 85 (17.5%), and 43 (8.9%) cases during the first trimester, the second trimester, the third trimester, 1 week postpartum, 6 months postpartum, and 12 months postpartum, respectively. During the prenatal period, factors such as monthly income, employment status, marital status, and thyroid function significantly impacted depression. Additionally, factors including monthly income, employment status, marital status, parity, and unintended pregnancy were found to affect the likelihood of developing postpartum depression. XGBoost was chosen for its accuracy (0.9097) and precision (0.9005) in predicting prenatal depression, as well as for its accuracy (0.9253) and precision (0.9523) in predicting postpartum depression.

**Discussion:**

In conclusion, the incidence of depression varies throughout the perinatal period, with different factors influencing prenatal and postpartum depression.

## Background

Perinatal depression (PND) encompasses depressive episodes experienced by women during pregnancy and up to 1 year after delivery (1). This clinically relevant disorder affects an estimated 15%–20% of women in China (2). The occurrence of PND varies across different stages of pregnancy. A comprehensive meta-analysis examining depression rates during pregnancy from 21 centers indicated that the incidence of depression in the first trimester ranged from approximately 2.2% to 12.6%, the second trimester exhibited rates between 10.7% and 14.8%, and rates of depression during the third trimester ranged from 7.4% to 12.6% (3). Notably, around 70% of pregnant women experiencing depression reported moderate levels of severity (4).

Maternal depression has been consistently linked to adverse perinatal outcomes. These outcomes encompass an increased likelihood of poor adherence to medical care, compromised nutrition (either insufficient or excessive gestational weight gain), diminished interpersonal and financial resources, as well as heightened risks of smoking and substance abuse and their associated consequences (5, 6). In the postpartum period, persistent depression has been found to impede maternal recovery, impair breast milk production, reduce the intensity of contractions, and increase the risk of postpartum hemorrhage (7).

Women experiencing severe depressive disorder during the perinatal period may exhibit extremely negative thinking patterns and, when subjected to certain triggers, may even develop suicidal tendencies or harm their infants (8). The incidence of suicides resulting from postpartum depressive disorder is alarmingly high in economically underdeveloped countries, reaching up to 20%, while economically developed countries report rates ranging from 5% to 14% (9). This represents a significant cause of maternal mortality worldwide. Furthermore, the effects of maternal depression on newborns include an increased likelihood of preterm birth, low birth weight, higher rates of diarrhea and infectious diseases, and impaired cognitive development (10, 11). Additionally, maternal depression during pregnancy raises the risk of depression in offspring during adulthood, with a risk 4.6 times higher than that in the general population (12). It can also contribute to the development of depression in the spouse (13). Although maternal depression is prevalent and associated with significant social dysfunction, it is only recently that multiple and concurrent risk factors have been identified. Identifying and addressing this issue could potentially reduce the number of suicides among perinatal women experiencing depression, as well as mitigate the adverse effects of untreated maternal depression on the cognitive and behavioral development of their children.

Perinatal depression is a multifaceted condition believed to arise from interactions involving the neuroendocrine hypothalamic-pituitary-adrenal axis, genetics, epigenetics, and environmental and social factors (1). Prior research has identified various risk factors associated with PND. These include race, age, primiparity, unplanned pregnancy, poor physical health, a history of mental illness, low social support within the family, and marital disharmony (14–19). Currently, the diagnosis of PND primarily relies on comprehensive assessments encompassing medical history, psychiatric examination, psychological evaluation, and physical examination, as there are no specific laboratory markers available. Many symptoms of PND lack specificity, such as insomnia, irritability, and self-doubt regarding the adjustment to the role of being a new mother. Consequently, accurately detecting PND can be challenging. Therefore, employing scales and objective indicators to assess the risk of PND, identify influencing factors, and actively intervene to reduce the incidence of PND holds significant importance.

In China, the number of births in 2022 reached 9.56 million. With the recent implementation of the three-child policy, the population of pregnant women is expected to further expand. Consequently, there will likely be a rise in the number of women who are of advanced age and at a higher risk of developing PND, ultimately impacting the incidence of this condition. Thus, the main objective of this study is to determine the incidence of PND and identify its risk factors at various stages of the perinatal period, followed by establishing a model to predict the occurrence of PND.

## Materials and methods

### Subjects and study setting

The present study was a prospective investigation conducted at the Affiliated People’s Hospital of Ningbo University, located in Ningbo, China. The study period spanned from March 2019 to August 2021. Information regarding the study was provided by the attending obstetricians to eligible women with singleton pregnancies who were less than 14 weeks pregnant. Women who expressed interest in participating in the study reached out to the investigators via WeChat or phone, following which an information meeting was arranged. Prior to enrollment, all participants autonomously provided their informed consent by signing the clinical research consent form. The ethical review board of the Affiliated People’s Hospital of Ningbo University has granted approval for this study (Approval No. 2017024, Date: 25 July 2017). Based on the research objectives, with an expected incidence rate of 15%, a significance level of 0.05, and a power of 0.80, approximately 196 samples are needed for each of the prenatal and postnatal stages, totaling 392 samples. Considering a 10% loss to follow-up rate, the adjusted minimum sample size is 436.

### Characteristics of the participants

The participants in the study attended an initial meeting before reaching gestational week 14, during which they provided various data points, such as age, place of residence, educational background, job, income, and marital status. The inclusion criteria for women participating in this study were as follows: (1) aged 20 years and older, (2) within the first 14 weeks of pregnancy, (3) having a singleton pregnancy, (4) planning to receive prenatal examinations and give birth at the designated research hospital, (5) capable of independently completing the questionnaire, and (6) possessing complete clinical data. The exclusion criteria were as follows: (1) previously diagnosed with a mental illness, (2) this birth resulted in a premature baby, (3) having experienced a stressful incident within the past year, defined as a significant life event that could affect mental health, such as divorce, loss of a loved one, or serious illness, and (4) refusal to participate in the study.

### Procedure

This study identified four common aspects as risk factors for PND, including: (1) sociodemographic factors, such as age, educational background, occupation, place of residence, marital status, and economic status; (2) obstetric factors, such as the number of previous labors, planned pregnancy, gender and health status of the newborns, and pregnancy-related complications; (3) past medical history; and (4) support from social and family members. Apart from the delivery method, postpartum complications, newborn gender, newborn health status, and newborn feeding methods, all other relevant information was obtained during the initial information meeting attended by the participants.

We assessed the participants using the Edinburgh Postnatal Depression Scale (EPDS), which consists of 10 items. Each item is scored on a four-point scale ranging from 0 to 3. Following existing literature, we categorized depression into four types: ≤9 as normal, 10–13 as mild depression, 14–20 as moderate depression, and >20 as severe depression. The questionnaire survey was conducted at six different time points: during the first trimester (pregnancy < 14 weeks), second trimester (14 weeks ≤ pregnancy < 28 weeks), third trimester (pregnancy ≥ 28 weeks), 1 week postpartum, 6 months postpartum, and 12 months postpartum.

This study utilized a combination of network and field investigation methods. The questionnaires were assigned unique numbers by the investigators, and all the questionnaire data were thoroughly checked and processed by two researchers. To ensure data quality, a random sampling of 10% of the questionnaire information was independently reviewed by the researchers. The privacy of the participants was strictly safeguarded throughout the entire study.

### Statistical analysis

In our analysis of factors related to PND, we took into consideration the distinctions between prenatal and postpartum risk factors. Thus, we categorized our findings into prenatal depression and postpartum depression for a more detailed examination. Prenatal depression was defined as an EPDS score of 10 or higher at any point during pregnancy. Similarly, postpartum depression was characterized by a score of 10 or higher during the postpartum period. All manually processed questionnaires were double-checked for data quality. Firstly, we visually represented the incidence of PND across different demographic groups using bar charts. Subsequently, we constructed classification models using Extreme Gradient Boosting (XGBoost), Logistic Regression (LR), Random Forest (RF), and Support Vector Machine (SVM) to predict the incidence of PND. We compared the accuracy and precision of these models. Accuracy refers to how close a measurement is to the true or actual value. Precision, on the other hand, refers to the consistency or repeatability of measurements. Considering both accuracy and precision as evaluation metrics, we selected the optimal model for the classification prediction.

## Results

### Baseline characteristics

**FIGURE 1:**
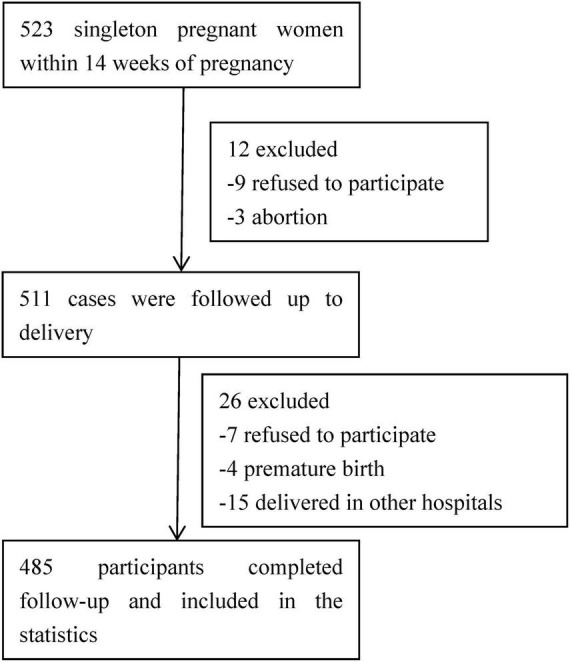
Flowchart of participants included in this study.

A total of 523 pregnant women with singleton pregnancies were initially included in the study. From March 2019 to August 2021, a total of 485 participants successfully completed all valid questionnaires. As illustrated in Figure 1, there were 38 participants who were subsequently excluded from the study. The reasons for exclusion include 16 women who declined to participate, 15 women who delivered in other hospitals, and 7 cases of abortion or premature delivery.

Table 1 provides an overview of the baseline characteristics. The study sample consisted of 485 pregnant women aged between 21 and 47 years, with an average age of 29 ± 6 years. The majority of participants were under 35 years old (70.52%) and were married (65.57%). A significant proportion had a higher level of education, with 59.80% having achieved a bachelor’s degree or above. In terms of residence, 58% reported living in urban or suburban areas. Among all participants, 31.34% identified as housewives, and 50.93% reported a monthly income below 5,000 CNY.

**TABLE 1:** The basic characteristics of the participants and the number of patients with prenatal and postpartum depression.

	*N* (%)	Prenatal depression *n* (%)*	Postpartum depression *n* (%)*
**Age (years)**			
<35	342 (70.52)	92 (0.27)	116 (0.34)
≥35	143 (29.48)	33 (0.23)	47 (0.33)
**Monthly income (CNY)**			
≤5,000	238 (49.07)	74 (0.31)	98 (0.41)
>5,000	247 (50.93)	49 (0.20)	64 (0.26)
**Education**			
Junior college and below	195 (40.20)	59 (0.30)	64 (0.33)
Bachelor degree	161 (33.20)	32 (0.20)	56 (0.35)
Master degree or above	129 (26.60)	34 (0.26)	43 (0.33)
**Employment status**			
Housewife	152 (31.34)	64 (0.42)	71 (0.47)
Non-housewife	333 (68.66)	60 (0.18)	93 (0.28)
**Place of residence**			
Town	282 (58.14)	73 (0.26)	93 (0.33)
Countryside	203 (41.86)	53 (0.26)	69 (0.34)
**Marital status**			
Unmarried	167 (34.43)	55 (0.33)	68 (0.41)
Married	318 (65.57)	70 (0.22)	95 (0.30)
**Cohabitation with spouse**			
Yes	249 (51.34)	62 (0.25)	77 (0.31)
No	236 (48.66)	61 (0.26)	85 (0.36)
**Parity**			
Primipara	205 (42.27)	55 (0.27)	78 (0.38)
Multipara	280 (57.73)	67 (0.24)	87 (0.31)
**Planned pregnancy**			
Yes	301 (60.06)	75 (0.25)	93 (0.31)
No	184 (37.94)	48 (0.26)	70 (0.38)
**Obesity or overweight**			
Yes	179 (36.91)	45 (0.25)	57 (0.32)
No	306 (60.09)	80 (0.26)	107 (0.35)
**Diabetes**			
Yes	152 (31.34)	41 (0.27)	50 (0.33)
No	333 (68.66)	73 (0.22)	117 (0.35)
**Thyroid function**			
Normal	338 (69.69)	78 (0.23)	122 (0.36)
Dysfunction	147 (30.31)	47 (0.32)	44 (0.30)
**Iron deficient anemia**			
Yes	75 (15.46)	20 (0.26)	26 (0.34)
No	410 (84.54)	98 (0.24)	131 (0.32)
**Mode of delivery**			
Spontaneous labor	381 (78.56)	–	133 (0.35)
Cesarean section	104 (21.44)	–	29 (0.28)
**Postpartum complications**			
Yes	173 (35.67)	–	66 (0.38)
No	312 (64.33)	–	100 (0.32)
**Newborn gender**			
Boy	275 (56.70)	–	91 (0.33)
Girl	210 (43.30)	–	71 (0.34)
**Newborn health status**			
Health	359 (74.02)	–	122 (0.34)
Need for medical monitoring	126 (25.98)	–	42 (0.33)
**Feeding patterns**			
Breast milk	218 (44.95)	–	50 (0.23)
Milk powder	107 (22.06)	–	39 (0.36)
Mixed feeding	160 (32.99)	–	61 (0.38)

*n*: If diagnosed with depression at any time during first, second, or third trimester, it was classified as prenatal depression; if diagnosed with depression at any time within 1 week, 6 months, or 12 months postpartum, it was classified as postpartum depression.

*The proportion of patients with depression within this group.

### Incidence rate of PND

In the present study, depression was observed in 75 (15.5%), 47 (9.7%), 25 (5.2%), 94 (19.4%), 85 (17.5%), and 43 (8.9%) cases among the total cohort of 485 pregnant women during the first trimester, second trimester, third trimester, 1 week postpartum, 6 months postpartum, and 12 months postpartum, respectively (Table 2). The incidence rate of prenatal depression in our study was 25.57% (124/485), while the incidence rate of postpartum depression was 33.81% (164/485). Notably, the incidence of depression varied at different time points. The highest proportion of severe depression was observed at 1 week postpartum (20.2%), followed by the first trimester (13.3%). Over the course of the study, the incidence of moderate depression declined gradually, while that of mild depression showed a gradual increase.

**TABLE 2:** The incidence rate of PND at six different time points.

	Total depression *N* (%)	Mild depression *N* (%)*	Moderate depression *N* (%)*	Severe depression *N* (%)*
First trimester	75 (15.5)	30 (40.0)	35 (46.7)	10 (13.3)
Second trimester	47 (9.7)	24 (51.1)	20 (42.6)	3 (6.4)
Third trimester	25 (5.2)	13 (52.0)	10 (40.0)	2 (8.0)
1 week postpartum	94 (19.4)	44 (46.8)	31 (33.0)	19 (20.2)
6 months postpartum	85 (17.5)	51 (60.0)	27 (31.8)	7 (8.2)
12 months postpartum	43 (8.9)	32 (74.4)	9 (20.9)	2 (4.7)

*The proportion of patients with different levels of depression among all depression patients within this time period.

### Prenatal depression related factors

**FIGURE 2:**
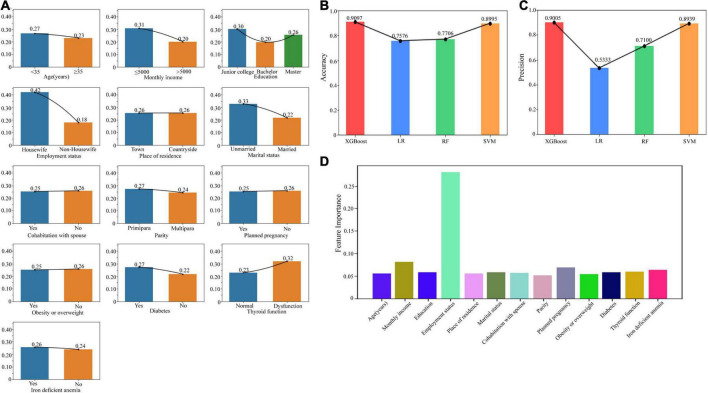
Risk factors related to prenatal depression. **(A)** The bar chart showed the proportions of individuals with prenatal depression across different demographic groups. **(B)** The accuracy of the models including XGBoost, LR, RF, and SVM. **(C)** The precision of the models including XGBoost, LR, RF, and SVM. **(D)** The feature weights of each risk factor based on the XGBoost model. XGBoost, Extreme Gradient Boosting; LR, Logistic Regression; RF, Random Forest; SVM, Support Vector Machine.

In the analysis of prenatal depression related factors, we included age, monthly income, education, employment status, place of residence, marital status, cohabitation with spouse, parity, planned pregnancy, obesity or overweight, diabetes, thyroid function, and iron deficient anemia. If diagnosed with depression at any time during the first, second, or third trimester, it was classified as prenatal depression. The number of patients was shown in Table 1. We presented the proportions of individuals with prenatal depression across different demographic groups using a bar chart (Figure 2A). The chart reveals that factors such as monthly income, employment status, marital status, and thyroid function have a significant impact on the likelihood of developing prenatal depression.

For construction of the classification models, we employed XGBoost, LR, RF, and SVM. The comparison of accuracy (Figure 2B) and precision (Figure 2C) is depicted in the chart below. Notably, the XGBoost model demonstrated the highest accuracy and precision, with scores of 0.9097 and 0.9005, respectively. Considering both accuracy and precision as evaluation metrics, we selected the XGBoost model for the classification prediction. The visualization of feature weights is displayed in Figure 2D. Among all the factors, employment status exhibited the highest influence on PND, followed by monthly income.

### Postpartum PND related factors

**FIGURE 3:**
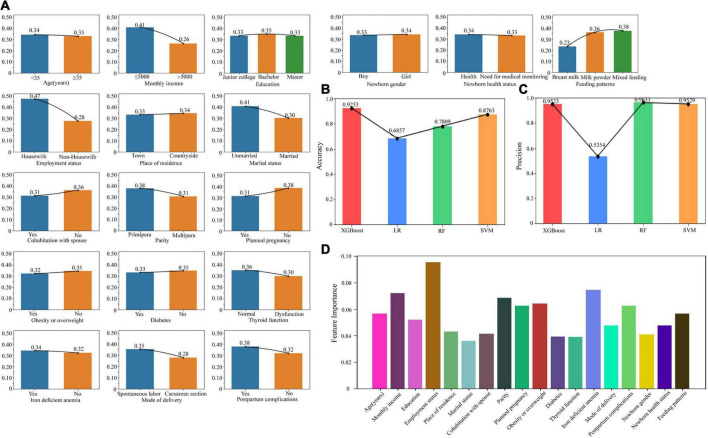
Risk factors related to postpartum depression. **(A)** The bar chart showed the proportions of individuals with postpartum depression across different demographic groups. **(B)** The accuracy of the models including XGBoost, LR, RF, and SVM. **(C)** The precision of the models including XGBoost, LR, RF, and SVM. **(D)** The feature weights of each risk factor based on the XGBoost model. XGBoost, Extreme Gradient Boosting; LR, Logistic Regression; RF, Random Forest; SVM, Support Vector Machine.

During the analysis of postpartum risk factors, we expanded the scope of our investigation compared to the prenatal phase by incorporating additional factors. These include mode of delivery, postpartum complications, infant gender, infant health status, and infant feeding methods. If diagnosed with depression at any time within 1 week, 6 months, or 12 months postpartum, it was classified as postpartum depression. The number of patients was shown in Table 1. To illustrate the distribution of postpartum depression across various demographic groups, we depicted the proportions of individuals affected in each group using a bar chart (Figure 3A). The chart explicitly demonstrates the significant impact that factors such as monthly income, employment status, marital status, parity, and unintended pregnancy have on the likelihood of developing postpartum depression.

We conducted a comparative study of four modeling methods, namely XGBoost, LR, RF, and SVM, and found that the XGBoost model outperformed the others in terms of accuracy (Figure 3B) and precision (Figure 3C). When employing the XGBoost model for our analysis, we discovered that employment status had the most significant impact on postpartum depression, followed by iron deficient anemia, monthly income, parity, obesity, and unintended pregnancy (Figure 3D).

## Discussion

Our study yields three key findings. Firstly, we observed variations in the incidence of depression across the six time points of perinatal period. And, the rate of PND is significantly higher postpartum compared to antepartum, reaching its peak in the first week after delivery. Secondly, there are differences in the related factors of prenatal and postpartum depression. Thirdly, work status emerged as a significant determinant in the occurrence of PND throughout the entire perinatal period.

Our study revealed a decline in the incidence of prenatal depression. The incidence rates of depression were approximately 15.5%, 9.7%, and 5.2% during the first, second, and third trimesters, respectively. Furthermore, the incidence of postpartum depression was observed to be 19.4%, 17.5%, and 8.9% at 1 week, 6 months, and 12 months postpartum. This could be attributed to female patients gradually adapting to their current circumstances. Therefore, greater attention should be paid to the mental health status of patients during early pregnancy and the postpartum period. In our study, the incidence rate of postpartum depression was observed to be 33.81%, surpassing the incidence rate of prenatal depression, which was 25.57%. This finding contradicts the research results of Gelaye et al. (20) who conducted a comprehensive analysis of 51 articles on antepartum depression and 53 articles on postpartum depression. They concluded that in low- and middle-income countries, the incidence of antepartum and postpartum depression was estimated at 25.8% (95% CI, 22.8%–29.0%) and 19.7% (95% CI, 16.9%–22.8%) respectively (20). However, it is important to acknowledge that these rates may vary based on geographical and socioeconomic factors.

Our study identified several significant predictors of prenatal depression, including being a housewife, a monthly income less than 5,000 CNY, being unmarried and thyroid dysfunction. Low household income has been identified as an important factor influencing prenatal depression. Previous research has shown that both hypo- and hyperthyroidism can lead to mood disorders, with clinical hypothyroidism being present in 1%–4% of patients with affective disorders (21). It is worth noting that abnormal thyroid function not only increases the risk of depression in the mother during pregnancy but also in their offspring (22). The underlying pathophysiology explaining the emotional and behavioral disorders in patients with hypothyroidism remains uncertain. However, it has been observed that nuclear receptors for triiodothyronine are present in the brain, particularly in regions such as the amygdala and hippocampus, which play a significant role in emotion regulation (22). Therefore, monitoring thyroid function during pregnancy is crucial. Previous study had shown that women with unintended pregnancies had higher odds of experiencing current stress, current depressive symptoms, and initiating prenatal care after the first trimester compared to women with intended pregnancies (23). However, in our research, there was no significant relationship between unintended pregnancies and prenatal depression. Furthermore, several studies have reported associations between gestational diabetes mellitus (GDM) and prenatal depression (24–26). Conversely, depression identified in early pregnancy may increase the risk of subsequent GDM development (26). However, in Lara-Cinisomo et al.’s (27) study, no significant association between prenatal depression and diabetes status was found. The same result was observed in our study.

In our study, several factors were found to significantly predict postpartum depression, including housewife status, monthly income less than 5,000 CNY, unmarried status, primipara, and unintended pregnancy. These findings are consistent with previous research that has identified various risk factors for postpartum depression, such as a history of mental illness, high living burden, poor socioeconomic status, adverse delivery outcomes, and postpartum sleep disturbances (20, 28). Notably, our study also revealed that unintended pregnancy was an important risk factor for postpartum depression, which is supported by Gastaldon et al.’s (29) study indicating a 50% increased risk of postpartum depression in women with unintended pregnancies. However, contrary to expectations, thyroid dysfunction was not found to be statistically significant in predicting postpartum depression, which aligns with the conclusions drawn by Keshavarzi et al. (30). Additionally, Tachibana et al.’s (19) study highlighted that antenatal risk factors, including “a perceived lack of family cohesion,” being a primipara, and receiving treatment for physical or psychiatric illness during pregnancy, could be utilized as indicators for predicting postpartum depression at 20 weeks. Furthermore, clinical studies have demonstrated that experiencing perinatal pain is associated with an increased risk of postpartum depression, while the use of epidural analgesia can potentially reduce this risk (31). Regrettably, these aspects were not included in our study.

In our study, being a housewife emerged as a significant factor influencing depression throughout the entire perinatal period. Across all six time points, the incidence of depression among housewives consistently exceeded that of non-housewives. This finding might be attributed to the demanding responsibilities of childcare that housewives undertake after childbirth. However, in another study by Tachibana et al. (19), “leaving or losing one’s job” was identified as a marginally significant predictor of postnatal depression among Japanese women. This discrepancy could potentially be attributed to differences in social contexts. In China, being a housewife often signifies long-term unemployment, lack of financial resources, limited social support, and lower family status.

In summary, each period of the perinatal period is associated with specific risk factors for depression. It is crucial to focus on different high-risk factors based on the patient’s gestational week and postpartum period to timely identify postnatal depression. This highlights the significance of our research.

However, our study has several limitations. Firstly, as mentioned earlier, we selected all eligible pregnant women from a single hospital, which may not fully represent the current situation in Ningbo, Zhejiang province, China. Additionally, we did not include various factors such as twins, premature infants, perinatal pain, laboratory examinations, and genetic factors. Women with a history of mental health problems are also a major limitation that compromises the generalization of the results. Previous research has confirmed that perinatal genes play a role as risk factors for postpartum depression (31). For example, one study identified DNA methylation in antenatal TTC9B and HP1BP3 genes, which can predict with 80% accuracy whether a woman will develop depression in the postpartum period (32, 33). Therefore, future research should encompass a comprehensive exploration of the risk factors of depression, incorporating symptoms, signs, laboratory tests, and relevant biological markers. Specifically, studies should aim to integrate genetic data, clinical information, and environmental factors to construct a more holistic understanding of the etiology of postpartum depression. By adopting a multi-faceted approach, future research has the potential to identify more accurate predictors of depression and develop targeted interventions to improve the mental health of mothers during the perinatal period.

## Conclusion

In summary, depression incidence varies across the perinatal period, with distinct factors affecting prenatal and postpartum depression. Employment status significantly influences PND rates. Our XGBoost model effectively predicts PND, offering a valuable tool for early detection and targeted intervention.

## Data Availability

The raw data supporting the conclusions of this article will be made available by the corresponding author, without undue reservation.
